# Normalized grip strength is inversely associated with DNAm age in middle age and older adults.

**DOI:** 10.1093/geroni/igab046.3511

**Published:** 2021-12-17

**Authors:** Mark peterson, Stacey Collins, Helen Meier, Jessica Faul

**Affiliations:** 1 University of Michigan, University of Michigan, Michigan, United States; 2 University of Michigan, Ann Arbor, Michigan, United States

## Abstract

There is a large body of evidence linking muscular weakness, as determined by low grip strength, to a host of negative aging-related health outcomes. Given these links, grip strength has been labeled a “biomarker of aging”; and yet, this metric provides no biological plausibility. The objective of this study was to determine the association between grip strength and DNA methylation (DNAm) age acceleration. Middle age and older adults from the 2006-2008 waves of the Health and Retirement Study with 8-years of follow-up were included. Cross sectional and longitudinal modeling were performed to examine the association between grip strength (normalized to body mass: NGS) and DNAm age acceleration, adjusting for cell composition, sociodemographic variables, and smoking. Three DNAm clocks were incorporated for estimating age acceleration including the established DunedinPoAm, Levine, and GrimAge clocks. There was a robust and independent cross sectional association between NGS and DNAm age for men (β:-0.36; p<0.001) and women (β:-0.36; p<0.001) using the DunedinPoAm clock, and for men only using the Levine (β:-8.04; p=0.01) and GrimAge (β:-4.76; p=0.01) clocks. There was also an independent longitudinal association between baseline NGS and DNAm age for men (β:-0.27; p<0.001) and women (β:-0.36; p<0.001) using the DunedinPoAm clock, and for women only using the Levine (β:-8.20; p<0.001) and GrimAge (β:-6.04; p<0.001) clocks. Our findings provide some evidence of age acceleration among men and women with lower NGS. Future research is needed to understand the extent to which DNAm age mediates the association between grip strength and chronic disease, disability, and mortality.

